# Supplementary material for: The tumour suppressor APC promotes HIV-1 assembly via interaction with Gag precursor protein
Source: Nat Commun. 2017 Jan 30;8:14259. doi: 10.1038/ncomms14259 (PMC5290283; doi:10.1038/ncomms14259)
Supplement: Supplementary Information — Supplementary Figures, Supplementary Tables and Supplementary References [file ncomms14259-s1.pdf]

Supplementary Figure 1

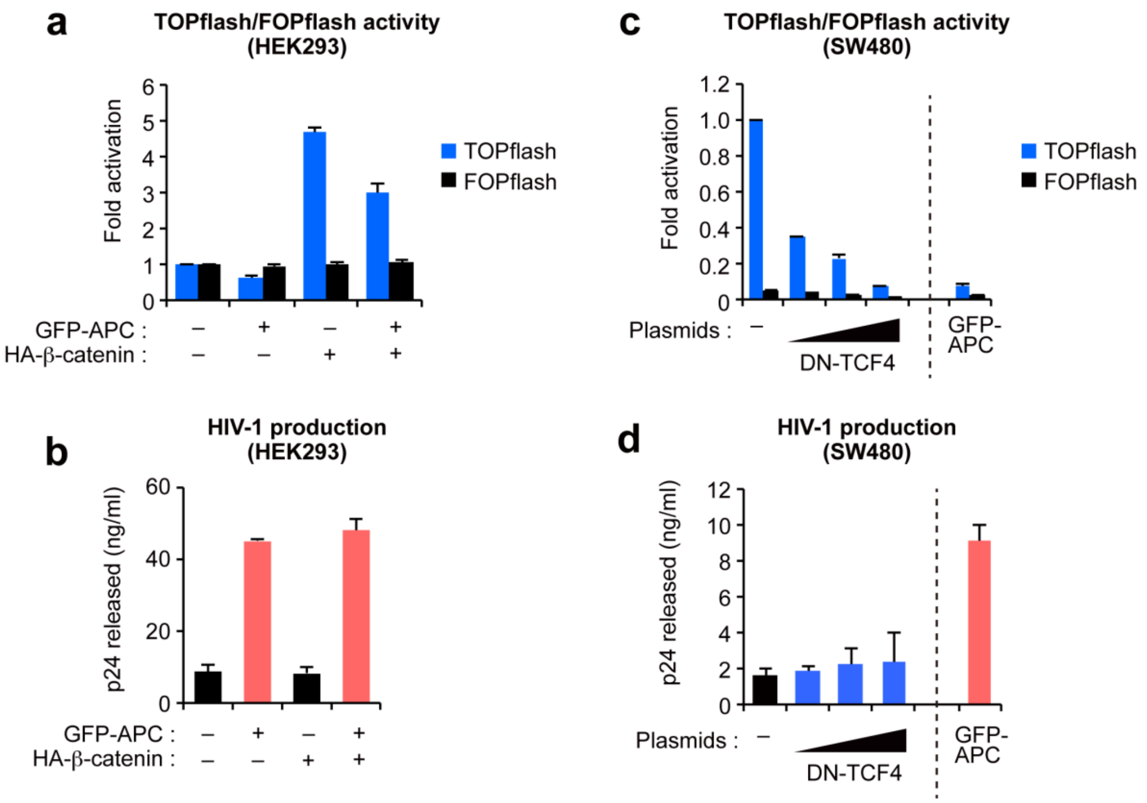

Supplementary Figure 1. Wnt/β-catenin signaling does not affect APC function in HIV-1 production.

(a, b) TOPflash (TCF4-luc) reporter assay (a) and HIV-1 production assay (b) of HEK293 cells expressing β-catenin and APC. FOPflash vector (TCF4-mut-luc) was also used as a negative control. HEK293 cells were co-transfected with pNL4-3 and GFP-APC in the presence or absence HA-tagged β-catenin. The bar chart in (b) indicates the viral p24 antigen levels in the culture supernatants. Note that the expression of β-catenin had no observable effects on APC-mediated HIV-1 production while it significantly increased TCF4-dependent transcriptional activity.

(c, d) SW480 cells were transfected with either TOPflash or FOPflash and co-transfected with different amounts of dominant-negative (dn)TCF4 or GFP-APC, followed by a gene reporter assay (c). SW480 cells were co-transfected with pNL4-3 together with different amounts of dnTCF4 or GFP-tagged APC. The bar chart in (d) indicates the viral p24 antigen levels in the culture supernatants. Note that the expression of dnTCF4 in SW480 cells (APC-mutated colorectal cancer cells) had no observable effects on the HIV-1 production, whereas it significantly reduced TCF4-dependent transcriptional activity. All graphs are presented as a mean ± s.d. ($n = 3$).

Supplementary Figure 2

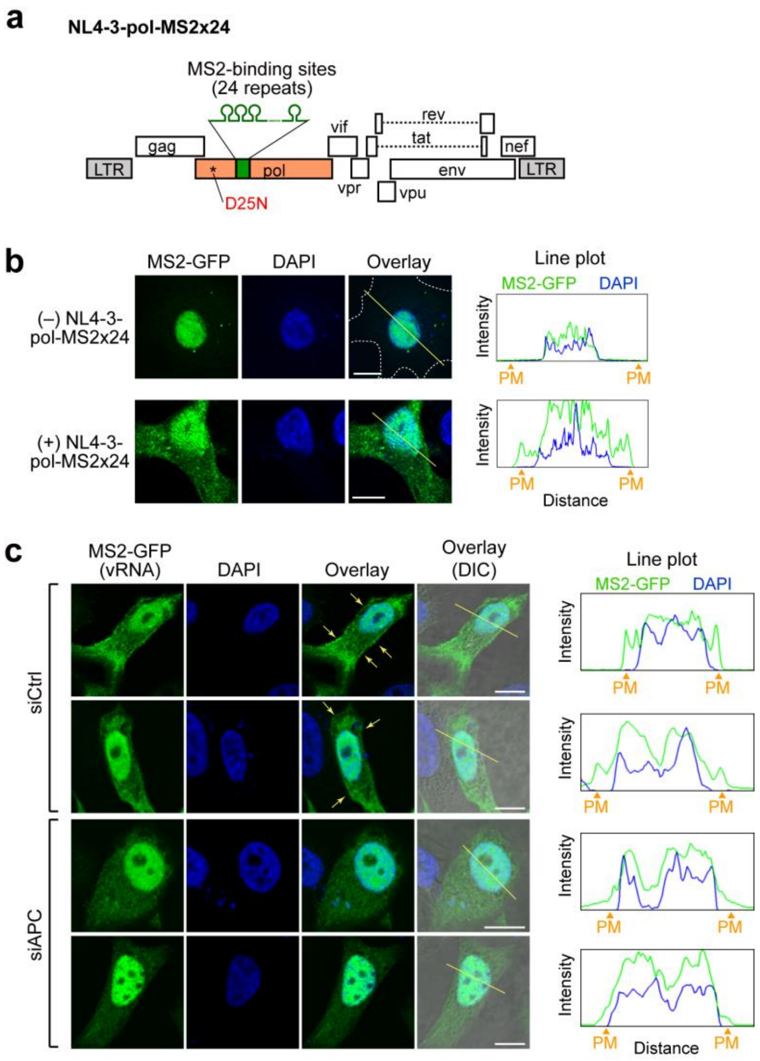

Supplementary Figure 2. vRNA labeling system used in this study.

(a) Schematic representation of the HIV-1 gene tagged with twenty-four repeats of MS2-binding sites (MBS) used in this study. MBS were inserted into a Pol gene with no protease activity due to a D25N mutation in the protease region.

(b) HeLa cells were co-transfected with the MS2-GFP expression plasmid with or without pNL4-3-pol-MS2x24. Cells were stained with anti-GFP antibody to highlight MS2-GFP signals, and the nuclei were counterstained with DAPI (blue). The dotted line denotes the cell border. Scale bar, 10 μm. Line plots in the right panels indicate the fluorescence intensity of MS2-GFP (green) and DAPI (blue) within regions of the plasma membrane (PM).

(c) Other representative example of cell images shown in Figure 6d.

Supplementary Figure 3

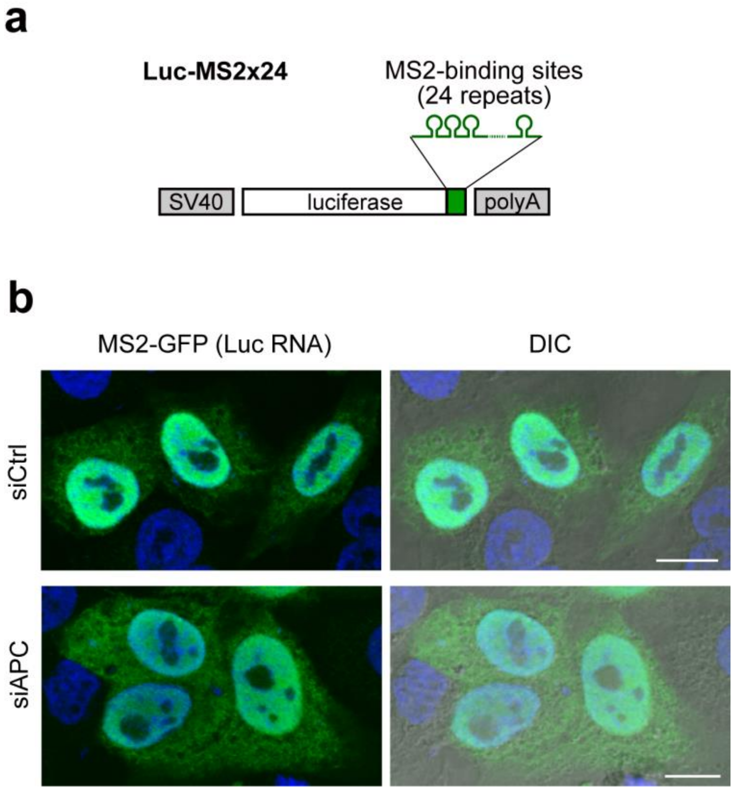

Supplementary Figure 3. APC does not affect non-retroviral RNA.

(a) Schematic representation of a non-viral (Renilla luciferase) RNA construct tagged with twenty-four repeats of MS2-binding sites (MBS). MBS were inserted at the end of luciferase gene of pGL4.73 (SV40-driven luciferase expression vector, obtained from Promega).

(b) HeLa cells were treated with control siRNA (siCtrl) or APC-targeted siRNA mix (siAPC) for 24 h prior to co-transfection with a pLuc-MS2x24 and MS2-GFP expression plasmid. Cells were stained with anti-GFP antibody and with DAPI (blue). Scale bar, 10 μm.

Supplementary Figure 4

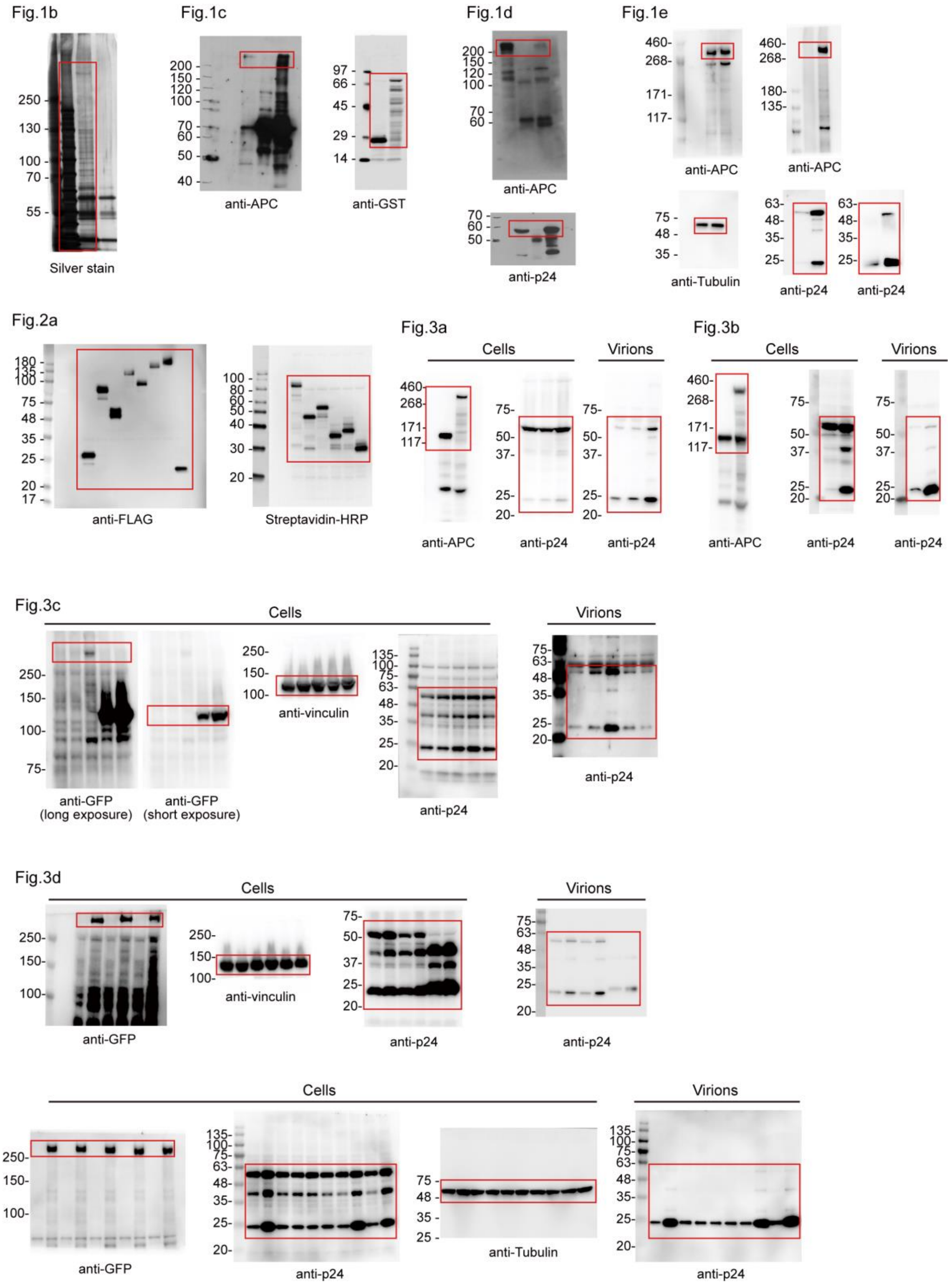

Supplementary Figure 4. Full images of the immunoblots presented in Figures 1-3.

Supplementary Figure 5

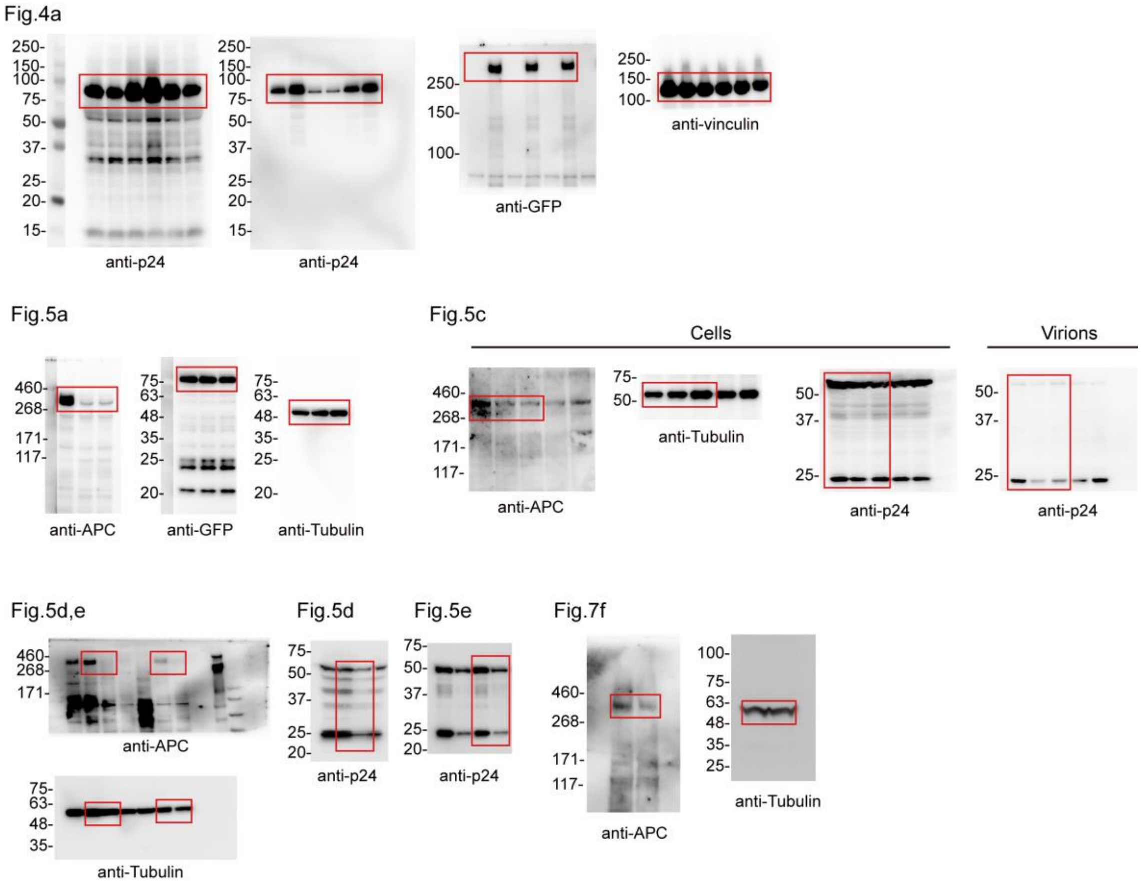

Supplementary Figure 5. Full images of the immunoblots presented in Figures 4-7.

Supplementary Table 1

Gel slice [a]	Gene symbol	M.W. [b]	Protein score [c]	Protein matches [d]	Peptide matches [e]	Previously reported HIV interactions [f]	Reference
**#1**	**APC**	**311.6**	**20**	**7**	**1**	–	
	LAMA3	366.7	17	6	2	Env, Tat	
**#2**	**NUMA1**	**238.1**	**614**	**44**	**18**	Gag-Pol, Tat	1
	MYH10	228.9	76	16	2	Gag, Tat	2
	BAZ2A	211.1	44	13	3	Vif	
	SEC16A	233.5	33	11	1	–	
	PARP12	226.3	19	4	1	–	
**#3**	**CLTC**	**191.5**	**674**	**32**	**24**	Gag, Pol, Env, Vpr	3
	CHD1	196.6	66	15	4	–	
	NUP188	196.0	62	7	3	–	
	ARMS	196.4	41	6	2	Tat	
	DNMT1	183.1	38	12	3	Tat	
	TAB182	181.7	26	16	3	–	
**#4**	**AMOT**	**118.0**	**1039**	**66**	**41**	Gag	4
	UBAP2L	114.5	313	10	9	Gag	2
	NAT10	115.7	275	21	12	Gag, Rev	2, 5
	DHX36	114.7	88	5	3	Rev	
	HLTF	113.9	81	9	4	Nef	
	CNTN1	113.3	49	5	2	–	
	DDX46	117.4	24	8	1	Tat	
	PSD3	116.0	23	8	1	–	
	RNF20	116.0	21	5	2	Gag, Env, Nef	3
**#5**	**HSP90B1**	**92.4**	**62**	**4**	**2**	–	
	MOGS	91.9	39	4	2	Gag, Pol, Env, Vpr, Nef	6
	KANK2	91.2	29	2	1	–	
	MCM6	92.8	25	4	1	–	
	AMPD3	88.8	16	7	1	–	
	ZNF616	90.3	21	10	1	–	

[a]Gel slice numbers indicated in Figure 1b. [b]Molecular weight (kD).
[c]MASCOT protein score. [d]Number of assigned spectra.
[e]Total numbers of peptides sequenced.
[f]Data collected from the HIV-1 Human Interaction Database (www.ncbi.nlm.nih.gov/genome/viruses/retroviruses/hiv-1/interactions/).

Supplementary Table 1. Gag-associated proteins identified by mass spectrometry.

Supplementary Table 2

Plasmids	Gene/Insert	References
pcDNA3-Gag-TAP	Gag(huOpt*)-TAP	See below
pCI-Gag-FLAG	Gag(huOpt)-FLAG	See below
pEGFP-N-Gag	Gag(huOpt)-FLAG-GFP	See below
pGEX-Gag	Gag(huOpt)-GST	7
pHTC-Gag	Gag(huOpt)-FLAG-HaloTag	See below
pNLF1-C-Gag	Gag(huOpt)-FLAG-NanoLuc	See below
pNL4-3	All HIV-1 proteins	8
pNL4-3/Fyn(10)fullMA	Fyn(10)**-Gag and the other viral proteins	9
pNL4-3/Fyn(10)ΔMA	Fyn(10)-Gag(MA-deficient) and the other viral proteins	9
pNL4-3/Fyn(10)fullMA/Gag-YFP	Fyn(10)-Gag-YFP, Tat, Rev and Nef	9, 10
pNL4-3/Fyn(10)-Gag(6A2T)-YFP	Fyn(10)-Gag(6A2T)-YFP, Tat, Rev and Nef	11
pNL4-3/Fyn(10)-Gag(RKswitch)-YFP	Fyn(10)-Gag(RKswitch)-YFP, Tat, Rev and Nef	11
pNL4-3/Gag-EGFP	Gag-EGFP (ΔPol) and the other viral proteins	12

*Human codon-optimized. **Fyn(10): N-terminal 10 amino acid derived from Fyn kinase.

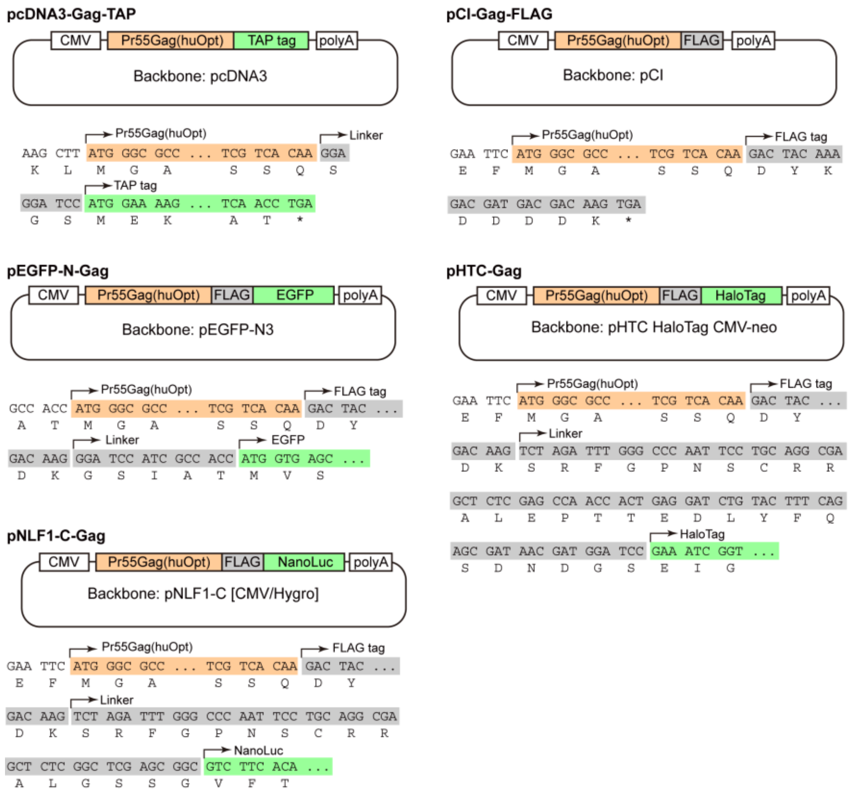

Supplementary Table 2. Gag fusion constructs and HIV-1 molecular clones used in this study.

Supplementary Table 3

Antibodies/Reagents	Source (Catalog number)	Dilution
APC	Santa Cruz Biotechnology (#sc-896)	1:100 (IF)
APC	Abcam (#ab58)	1:1000 (WB)
α-Tubulin	Sigma-Aldrich (#T6199)	1:10000
Vinculin	Sigma-Aldrich (#V9264)	1:1000
HA	Roche (#11867423001)	1:1000
FLAG	Sigma-Aldrich (#F3165)	1:10000
GST	Santa Cruz Biotechnology (#sc-138)	1:10000
GFP	Wako (#012-20461)	1:100 (IF)
GFP	Roche (#11814460001)	1:1000 (WB)
HIV-1 Gag p24	NIH AIDS Reagent Program (#3537)	1:1000
Streptavidin-HRP Conjugate	GE Healthcare (#RPN1231)	1:5000
HRP-conjugated anti-mouse IgG	GE Healthcare (#NA931)	1:10000
HRP-conjugated anti-rabbit IgG	GE Healthcare (#NA934)	1:10000
Cholera Toxin Subunit B, Alexafluor594-conjugated	Thermo Fisher Scientific (#C34777)	1:200

Supplementary Table 3. Antibodies and reagents used in this study.

Supplementary References

1. Gautier VW, Gu L, O'Donoghue N, Pennington S, Sheehy N, Hall WW. In vitro nuclear interactome of the HIV-1 Tat protein. *Retrovirology* **6**, 47 (2009).
2. Engeland CE, *et al.* Proteome analysis of the HIV-1 Gag interactome. *Virology* **460-461**, 194-206 (2014).
3. Milev MP, Ravichandran M, Khan MF, Schriemer DC, Mouland AJ. Characterization of staufen1 ribonucleoproteins by mass spectrometry and biochemical analyses reveal the presence of diverse host proteins associated with human immunodeficiency virus type 1. *Frontiers in microbiology* **3**, 367 (2012).
4. Mercenne G, Alam SL, Arii J, Lalonde MS, Sundquist WI. Angiomotin functions in HIV-1 assembly and budding. *eLife* **4**, (2015).
5. Le Sage V, Cinti A, Valiente-Echeverria F, Mouland AJ. Proteomic analysis of HIV-1 Gag interacting partners using proximity-dependent biotinylation. *Virology journal* **12**, 138 (2015).
6. Jager S, *et al.* Global landscape of HIV-human protein complexes. *Nature* **481**, 365-370 (2012).
7. Ryo A, *et al.* SOCS1 is an inducible host factor during HIV-1 infection and regulates the intracellular trafficking and stability of HIV-1 Gag. *Proceedings of the National Academy of Sciences of the United States of America* **105**, 294-299 (2008).
8. Adachi A, *et al.* Production of acquired immunodeficiency syndrome-associated retrovirus in human and nonhuman cells transfected with an infectious molecular clone. *Journal of virology* **59**, 284-291 (1986).
9. Chukkapalli V, Hogue IB, Boyko V, Hu WS, Ono A. Interaction between the human immunodeficiency virus type 1 Gag matrix domain and phosphatidylinositol-(4,5)-bisphosphate is essential for efficient gag membrane binding. *Journal of virology* **82**, 2405-2417 (2008).
10. Hogue IB, Hoppe A, Ono A. Quantitative fluorescence resonance energy transfer microscopy analysis of the human immunodeficiency virus type 1 Gag-Gag interaction: relative contributions of the CA and NC domains and membrane binding. *Journal of virology* **83**, 7322-7336 (2009).
11. Llewellyn GN, Grover JR, Olety B, Ono A. HIV-1 Gag associates with specific uropod-directed microdomains in a manner dependent on its MA highly basic region. *Journal of virology* **87**, 6441-6454 (2013).
12. Haraguchi H, Sudo S, Noda T, Momose F, Kawaoka Y, Morikawa Y. Intracellular localization of human immunodeficiency virus type 1 Gag and GagPol products and virus particle release: relationship with the Gag-to-GagPol ratio. *Microbiology and immunology* **54**, 734-746 (2010).
